# Surface-Engineered MoO_x_/CN Heterostructures Enable Long-Term SF_6_ Photodegradation via Suppressed Fluoridation

**DOI:** 10.3390/molecules30071481

**Published:** 2025-03-27

**Authors:** Wenhui Zhou, Boxu Dong, Ziqi Si, Yushuai Xu, Xinhua He, Ziyi Zhan, Yaru Zhang, Chaoyu Song, Zhuoqian Lv, Jiantao Zai, Xuefeng Qian

**Affiliations:** Shaoxing Research Institute of Renewable Energy and Molecular Engineering, School of Chemistry and Chemical Engineering, Shanghai Jiao Tong University, Shanghai 200240, China; zhouwenhui1995@sjtu.edu.cn (W.Z.); kingjames@sjtu.edu.cn (B.D.); siziqi520@163.com (Z.S.); yushuai-xu@sjtu.edu.cn (Y.X.); xinhuahe@sjtu.edu.cn (X.H.); joieyzhan@gmail.com (Z.Z.); fishzyrrr@sjtu.edu.cn (Y.Z.); songchaoyu@sjtu.edu.cn (C.S.); lzq323@sjtu.edu.cn (Z.L.)

**Keywords:** photocatalysis, sulfur hexafluoride degradation, stability, heterostructures

## Abstract

Sulfur hexafluoride (SF_6_), the strongest greenhouse gas, has great challenges in degradation because of its stable structure, posing significant environmental concerns. Photocatalysis offers an environmentally friendly, low-energy solution, but the fluoride deposition on catalysts reduces their activity, thus limiting their large-scale application. To prevent catalyst fluoride poisoning, we report a thin-layer graphitic carbon nitride (CN) material loaded with MoO_x_ (CNM) that resists fluoride deposition for long-term SF_6_ degradation. By combining molecular structure design and nanostructure regulation, we construct a photocatalyst with enhanced charge carrier mobility and reduced transport distances. We find that the CNM exhibits a high specific surface area, increased contact between reactants and active sites, and efficient electron–hole separation due to the Mo-N bonds, achieving an SF_6_ degradation efficiency of 1.73 mmol/g after one day due to the prolonged catalytic durability of the catalyst, which is eight times higher than pristine g-C_3_N_4_ (0.21 mmol/g). We demonstrate the potential of CNMs for low-energy, high-efficiency SF_6_ degradation, offering a new approach to mitigate the environmental impact of this potent greenhouse gas. We envision that this study will inspire further research into advanced photocatalytic materials for environmental remediation, contributing to global efforts in combating climate change.

## 1. Introduction

Sulfur hexafluoride (SF_6_) plays a crucial role in various fields such as the electrical and aerospace industries [1,2,3,4,5,6]. However, it is also an extremely potent greenhouse gas [7], with a global warming potential (GWP) 24,300 times that of an equivalent amount of CO_2_ [8]. Therefore, the degradation of SF_6_ has become an urgent task. The stable structure of SF_6_ makes it difficult to degrade [9,10,11]. Photocatalysis, which utilizes solar energy to degrade pollutants, is an environmentally friendly and low-energy-consumption method for degrading stable SF_6_ [12,13]. Although there have been reports of improvements in the reaction system, the large-scale development of photocatalytic technology has been limited by the lack of efficient photocatalysts [14,15,16,17]. Hence, the development of photocatalysts is the focal point of photocatalytic technology advancements.

For photocatalytic degradation of sulfur hexafluoride (SF_6_), although our previous work has shown that the gas–liquid–solid three-phase system can improve the performance of the catalyst, the photocatalyst still faces the issue of deactivation in 6 h due to fluorine poisoning [12]. Thus, the reaction between the photocatalyst and inorganic F ions should be inhibited to develop photocatalyst performance. Organic polymers are less likely to be covered by inorganic fluoride in the solution and thus can keep their activity, due to their different hydrophobic/hydrophilic properties. Recently, two-dimensional conjugated polymer semiconductors, particularly covalently bonded carbon nitride materials (g-C_3_N_4_), have emerged as highly promising and representative materials [18,19,20,21,22,23]. Owing to their high chemical and thermal stability, visible-light response, and simple preparation methods, they have garnered global attention in artificial photosynthesis. Compared with traditional bulk materials, thin-layer carbon nanosheets offer significant advantages in enhancing photocatalytic activity; for instance, they possess larger surface areas, more exposed active sites, and shorter charge carrier transport distances from the bulk to surface-active sites. Moreover, forming porous structures on two-dimensional carbon nanosheets can further significantly improve charge and mass transport during the photocatalytic process [24].

However, g-C_3_N_4_, due to the absence of metal, lacks efficient active sites for the adsorption and activation of sulfur hexafluoride. Despite this, it not only facilitates the adsorption of organic reactants but also boasts a large specific surface area. Additionally, molybdenum fluoride exhibits a higher susceptibility to hydrolysis into MoO_x_ in solution due to thermodynamic factors [25]. So, when MoO_x_, which contains variable-valence metals, acts as active centers, it is less likely to be poisoned and deactivated by fluoride ions [17,26]. In addition, Mo-based inorganic nanomaterials have demonstrated excellent catalytic performance and favorable characteristics for electron and hole transport, showing great application potential in photocatalysis. Research has shown that MoO_2_ has extensive applications in photocatalysis, including selective oxidation of lactic acid, CO_2_ photocatalytic reduction, and photocatalytic ammonia synthesis [27,28,29,30]. Combining high specific surface area materials that favor organic reactants with Mo-based inorganic nanocatalytic materials with variable metal valences is expected to provide an efficient pathway for the efficient adsorption and degradation of SF_6_.

Here, we synthesized a MoO_x_-loaded thin-layer carbon nitride composite (CNM), which enhanced the photocatalyst’s specific surface area, facilitating reactant contact with MoO_2_ active sites and boosting SF_6_ degradation. The organophilic g-C_3_N_4_ substrate improved SF_6_ mass transfer in organic solvents and sacrificial agent binding. Adsorption tests, DFT calculations, and in situ IR confirmed MoO_2_’s role in SF_6_ adsorption/activation. XPS revealed Mo-N bonds between MoO_x_ and C_3_N_4_, establishing an electron transport channel that significantly enhanced charge separation. This design achieved SF_6_ degradation efficiency >10× higher than previously reported and surpassed our prior work due to prolonged catalyst durability. The findings provide a novel strategy for low-energy, stable, and efficient SF6 degradation.

## 2. Results and Discussion

### 2.1. Synthesis and Structure Characterization of Layered CNM Samples

**Figure 1:**
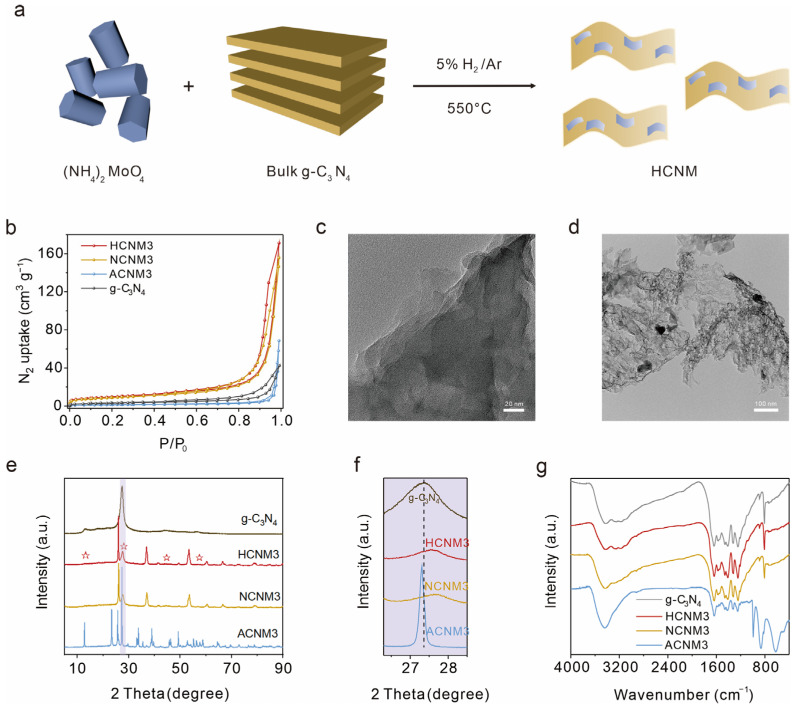
Synthesis and structure characterization of g-C_3_N_4_ and layered CNM: (**a**) schematic illustration of HCNM nanocomposite synthesis by in situ growth of MoO_x_ on the g-C_3_N_4_, (**b**) N_2_ sorption isotherms at 77 K, the transmission electron microscope (TEM) image of (**c**) g-C_3_N_4_ and (**d**) HCNM3, (**e**) X-ray diffraction (XRD), where ☆ represents g-C_3_N_4_, (**f**) zoom-in of the purple area in the total spectrum (**e**), and (**g**) FTIR spectra of g-C_3_N_4_ and CNM.

As depicted in Figure 1a, layered CNM samples were synthesized via a straightforward two-step high-temperature calcination process. Initially, melamine underwent high-temperature polymerization to yield bulk g-C_3_N_4_. Chemically active (NH_4_)_2_MoO_4_ was employed as the Mo source, serving as the metal site for the adsorption and activation of sulfur hexafluoride (SF_6_). Subsequently, through additional calcination of the mixture of g-C_3_N_4_ and (NH_4_)_2_MoO_4_ under varying gas atmospheres, g-C_3_N_4_ underwent re-polymerization and functionalization with MoO_x_ groups, culminating in the target photocatalyst CNM. Nitrogen adsorption–desorption experiments indicated that the pore volumes of HCNM3, NCNM3, and ACNM3 reach up to 0.26 cm^3^/g, 0.23 cm^3^/g, and 0.10 cm^3^/g, respectively, surpassing that of g-C_3_N_4_ (0.06 cm^3^/g) (Figure 1b). The specific surface areas of HCNM3 and NCNM3 are 34.5 m^2^/g and 30.7 m^2^/g, respectively, which are greater than that of g-C_3_N_4_ (10.86 m^2^/g), attributable to the gas etching effect and the high-temperature corrosion effect of the molybdenum salt. Unfortunately, the specific surface area of ACNM3 calcined in air is relatively small (4.60 m^2^/g). Moreover, the morphology of HCNM3 was characterized by scanning electron microscope (SEM, Figure S1) and transmission electron microscope (TEM, Figure 1c,d) images. It can be observed that HCNM3 exhibits a thinner sheet-like structure compared to g-C_3_N_4_. From the SEM images, CNM is revealed to be a layered material. The surface of HCNM3 is no longer smooth, and in-plane holes can be discerned. The G-C_3_N_4_ sample displays stacked sheets with a transition from polycrystalline to quasi-crystalline (Figure S2). However, as shown in Figure S3, the apparent porous nanosheets with some wrinkles are observed for HCNM3. Particles of approximately 20–30 nm in size can be detected, possessing an estimated d-spacing of 0.34 nm, which is attributed to the interplanar distance between the (110) planes of monoclinic MoO_2_ and is in good agreement with our subsequent XRD results. The element mapping images (Figure S3c,d) reveal the aggregation of Mo and O on these small particles, further confirming that these nanoparticles are oxides of molybdenum. Clearly, the high surface area of CNMs can be primarily attributed to its abundant pores and unique ultrathin morphology. Such a high specific surface area is of great significance for enhancing the catalytic properties of the materials.

X-ray powder diffraction (XRD) patterns were utilized to ascertain the crystalline phases of the bulk g-C_3_N_4_ and CNM samples. As illustrated in Figure 1e,f and Figure S4, the HCNM and NCNM composites exhibit superimposed XRD patterns characteristic of g-C_3_N_4_ and MoO_2_ (JCPDS: 32-0671), while the ACNM samples additionally display a pronounced MoO_3_ diffraction peak (JCPDS: 50-0508), indicating the presence of heterostructures within the composites [31,32,33,34,35]. The peak around 27°, corresponding to the (002) π–π interlayer stacking of the conjugated aromatic system in g-C_3_N_4_, exhibits a positive shift and reduced intensity, which suggests that the interlayer stacking distance has been diminished due to enhanced van der Waals attraction between adjacent heptazine layers and the reduced thickness of the layered carbon nitride crystals [36,37].

The chemical structures of CNMs and g-C_3_N_4_ were confirmed through the combined application of Fourier transform infrared (FTIR) and Raman spectroscopy. In the FTIR spectra (Figure 1g and Figure S5), all samples exhibit a sharp peak at 812 cm^−1^, which corresponds to the characteristic breathing mode of the heptazine heterocyclic ring. The absorption band in the 1100–1700 cm^−1^ range is also attributed to the tri-s-triazine main structural unit. The broad peak located at 3000–3600 cm^−1^ is due to the stretching vibration of the –NH_x_ groups of melamine. Additionally, the ACNM catalysts, which contain a higher proportion of Mo, display a new absorption peak at 985 cm^−1^ compared to g-C_3_N_4_, which is assigned to the symmetric vibration of the NC_2_ bond in the metal–NC_2_ group [38]. Peaks in the 400–900 cm^−1^ range were attributed to the intermediate bridging O–Mo–O bond, Mo=O, and Mo–O bonding types of MoO_3_ [39,40]. Raman spectra (Figure S6) similarly detect signal peaks for MoO_x_ and g-C_3_N_4_ in the CNM samples [41,42,43,44,45,46,47]. The MoO_x_ peaks in HCNMs are weaker, yet there are strong MoO_x_ diffraction peaks in the XRD, which may be due to the metal particles obtained in the hydrogen–argon mixed gas being dispersed on the polymer substrate and coordinated with the polymer through Mo-N bonds.

### 2.2. Chemical Structural Analysis

**Figure 2:**
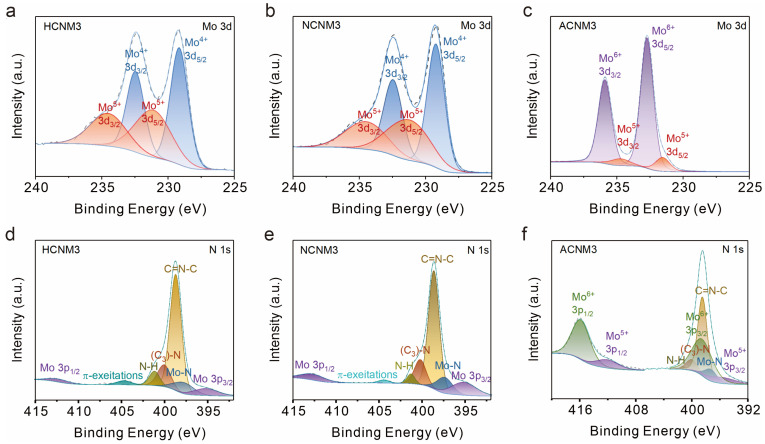
X-ray photoelectron spectroscopy (XPS) analysis of HCNM3, NCNM3, and ACNM3: (**a**–**c**) Mo 3d of samples, (**d**–**f**) N 1s core level of samples.

CNMs obtained under different atmospheres may possess catalysts with different redox states. X-ray photoelectron spectra (XPS) were employed to characterize CNMs prepared under various atmospheres and at different ratios (Figure 2 and Figures S7–S10) to analyze the elemental valence state and chemical composition of CNM catalysts. The wide-scan XPS confirm the presence of Mo, O, N, and C in CNMs and O, N, and C in g-C_3_N_4_. All binding energies in the XPS spectra have been calibrated using the C 1s peak at 284.8 eV. As shown in Figure 2a,b, and Figure S7, for samples synthesized under H_2_/Ar and N_2_ gas, the Mo 3d core-level spectrum consists of a spin–orbit doublet with peaks at 232.5 and 229.3 eV, attributed to the 3d_3_/_2_ and 3d_5_/_2_ of Mo_4_^+^ species. Additionally, two peaks at 234.5 and 231.2 eV binding energies can be assigned to Mo^5^^+^ [40,48]. For the air-calcined sample ACNM3 (Figure 2c), peaks located at 235.8 and 232.7 eV correspond to Mo cations in high oxidation states (Mo^6^^+^), and other peaks at 234.5 and 231.4 eV are attributed to the Mo(V) oxidation state. The presence of Mo(V) could be due to the reduction by –NH_2_ groups of g-C_3_N_4_ during sample preparation.

The high-resolution XPS spectrum of the N 1s peak is shown in Figure 2d–f and Figure S8. Both CNMs and g-C_3_N_4_ include sp^2^-bonded nitrogen in C–N=C (398.7 eV), nitrogen in tertiary N–(C)_3_ groups (400.3 eV), and the presence of amino groups (C–N–H, 401.3 eV) caused by imperfect polymerization. Compared to g-C_3_N_4_, CNMs have a higher proportion of bridging nitrogen ((C)_3_–N), indicating the re-polymerization of g-C_3_N_4_ during the secondary calcination process. Moreover, in ACNM3, the N–H bonding content is significantly reduced, indicating that –NH_2_ has been oxidized. The analysis of the π-excitation peak at 404.4 eV in CNMs reveals that an excess of Mo leads to a weakening of π–π interactions within g-C_3_N_4_, with the peak at 404.4 eV being almost undetectable in the ACNM3 sample. It can be observed that the N 1s peak overlaps with the Mo 3p peak. The characteristic peak located at a binding energy of approximately 395.3 eV is attributed to Mo 3p_3/2_, which is about 17.2 eV lower than the peak position of 3p_1/2_ at around 412 eV. Additionally, CNMs show an extra peak corresponding to the Mo–N bond at 397.8 eV compared to g-C_3_N_4_ (Figure S8c), indicating the coordination of Mo to N within the g-C_3_N_4_ framework [49,50]. Notably, the deconvolution results of HCNM1 show a significant proportion of Mo–N at 397.8 eV [51,52] (Figure S8a), and HCNMs synthesized under H_2_/Ar atmosphere contain more Mo–N than NCNM3 synthesized under N_2_ gas, suggesting that MoO_x_ particles are smaller and more uniformly dispersed on g-C_3_N_4_ under a hydrogen atmosphere, which also explains the stronger peaks belonging to Mo–O bonds observed in the Raman spectra of NCNM3 (Figure S6).

The deconvolution of C 1s for g-C_3_N_4_ exhibited three peaks at 284.8, 286.7, and 288.2 eV, corresponding to C–C/C=C, C–O, and C–NH_x_, respectively (Figure S9) [53]. The analysis of the C 1s spectra of HCNMs reveals that when the molybdenum content is low, the C–O peak is absent, indicating that the polymer substrate is reduced by H_2_/Ar. As the Mo content increases, a peak at 288.8 eV appears and gradually intensifies, attributed to C–OOH [54], indicating that an excess of molybdenum salts can oxidize the C-based substrate, even under reducing atmospheres. For samples HCNM3, NCNM3, and ACNM3 with the same proportion of molybdenum salts added, as the reducibility of the calcination atmosphere decreases and the oxidizing nature increases, a greater proportion of C is oxidized. Table 1 shows the C/N atomic ratios of CNMs obtained from the same precursors under different atmospheres. As the oxidizing nature of the secondary calcination atmosphere increases and the reducibility decreases, the C/N ratio gradually increases.

Figure S10 shows the O 1s spectra, with peaks attributable to oxides and chemisorbed oxygen visible. Although the assignments of oxygen species are not yet well established, all peak positions are in good agreement with literature values: the main component peak at 532.6 eV (yellow curve) corresponds to oxygen species dissolved in the metal or to adsorbed oxygen; the peak at 530.8 eV (blue curve) is attributed to Mo–O; the peak located at the lower binding energy of about 529 eV (purple curve) corresponds to (CₙNₘ)–O, and peaks with the higher binding energy at about 534 eV (green curve) are assigned to surface strongly adsorbed oxygen (OH^−^ and O^−^) [55].

The XPS demonstrate that different molybdenum salt precursors and calcination atmospheres not only affect the oxidation state and concentration of MoO_x_ loaded on the material but also impact the polymer substrate. They also confirm that the material is a composite of MoO_x_ and C_3_N_4_ coordinated through Mo–N bonds.

### 2.3. Photocatalytic Performance Evaluation

**Figure 3:**
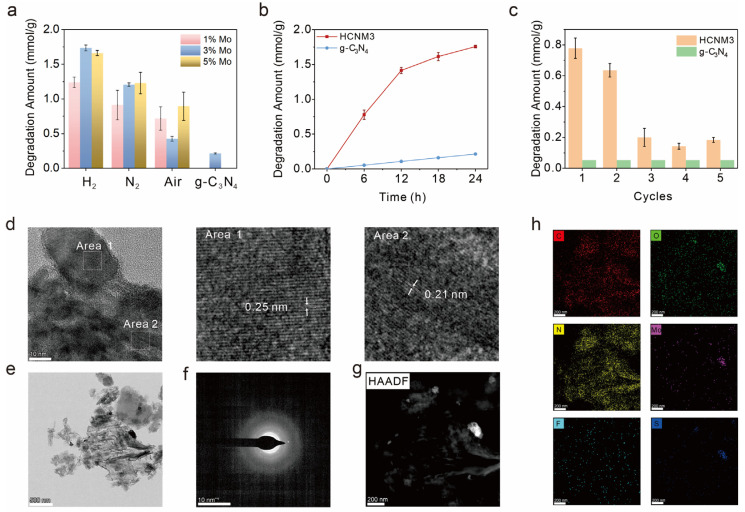
Photocatalytic performance and cyclic stability evaluation: (**a**) SF_6_ degradation of different samples under LED irradiation (λ = 365 nm) at room temperature, (**b**) kinetic curves of photoreduction SF_6_ degradation of HCNM3 and g-C_3_N_4_, (**c**) SF_6_ degradation amount in the stability tests of the HCNM3 and g-C_3_N_4_ samples with the reaction hours of 6 h, (**d**) HRTEM, (**e**) TEM, (**f**) selected area electron diffraction (SAED), (**g**) HAADF-STEM (scanning transmission electron microscope) images, and (**h**) STEM-EDS maps of HCNM3 after 24 h reaction.

We evaluated the photocatalytic performance of CNMs with different Mo loadings prepared under various atmospheres using an optimized system from our previous work for the degradation of SF_6_. As shown in Figure 3a, the photocatalytic activity of g-C_3_N_4_ for SF_6_ degradation was 0.21 mmol/g within one day, while the introduction of Mo species significantly enhanced the photocatalytic activity of CNMs. Overall, the HCNMs calcined under a hydrogen–argon atmosphere exhibited superior performance compared to the NCNMs obtained under nitrogen and the ACNMs obtained under air. Different Mo loadings affected the efficiency of catalytic SF_6_ degradation. The optimal CNM was HCNM3, achieving an SF_6_ degradation efficiency of 1.73 mmol/g, which is 8 to 9 times that of g-C_3_N_4_. Unfortunately, although HCNM3 demonstrates a longer operational duration compared to previous versions, it exhibits poor catalytic cycling stability (Figure S11). The temporal profile indicates that HCNM3 maintains relatively good stability within 12 h. However, after exceeding 12 h of operation, the degradation rate undergoes a significant decline, although it remains superior to C_3_N_4_ (Figure 3b). Based on previous findings where the catalyst undergoes fluoride poisoning-induced deactivation within 6 h in a three-phase system, we conducted cyclic experiments for a 6 h reaction (Figure 3c). The results reveal a partial activity loss in the third cycle, yet the catalytic activity remains largely preserved after three cycles and still surpasses that of untreated g-C_3_N_4_.

We collected and characterized the catalysts after the reaction; the IR and XRD signals showed no significant changes before and after the reaction (Figure S12). The morphology of the samples after the reaction was characterized by TEM/SEM (Figure 3d–g and Figure S13). No obvious morphological changes were observed in the SEM and TEM images. The high-resolution TEM images revealed lattice fringes of 0.21 nm and 0.25 nm, corresponding to the 1T-MoS_2_ and the (−211) lattice plane of MoO_2_, respectively [56,57]. MoS_2_ also serves as a photocatalyst. Although its incorporation reduces the catalytic degradation efficiency of HCNM3, it enhances the degradation of SF_6_ compared to C_3_N_4_. As shown in Figure 3h, the element mapping images revealed that the F element was uniformly distributed on the photocatalyst HCNM3 without significant aggregation, which may be attributed to the adsorption of fluorine species on the surface of the catalyst. The S element mainly accumulates on MoO_x_, indicating the interaction between MoO_x_ and sulfur ions in the solution. We also compared the XPS before and after the reaction (Figure S14). The results showed increased COOH signals on the carbon nitride substrate and a higher content of Mo(V), indicating a charge transfer process between the catalyst and the reactants. Additionally, deposition signal peaks for the S and F elements were also detected.

### 2.4. Mechanistic Insights into Enhanced Photocatalysis

**Figure 4:**
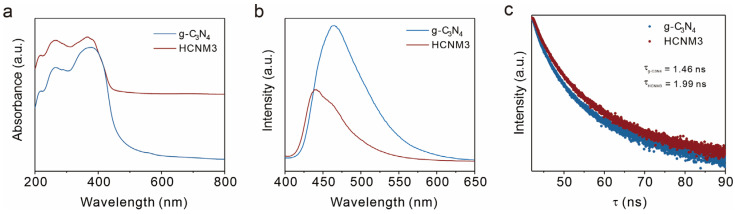
Photocatalytic properties of the samples, including light absorption and carrier behavior. (**a**) UV–vis DRSs of g-C_3_N_4_ and HCNM3, (**b**) room temperature (298 K) steady-state PL spectra of the g-C_3_N_4_ and HCNM3 samples, and (**c**) time-resolved fluorescence kinetics monitored at the corresponding emission peaks.

To gain insights into the mechanism behind the enhanced photocatalytic performance, the light absorption properties of the synthesized samples were examined using diffuse reflectance spectra (DRSs). As shown in Figure 4a, introducing Mo leads to a slight red-shift in the optical edges of the HCNM3 sample, demonstrating superior light absorption capabilities compared to the original g-C_3_N_4_. The color of g-C_3_N_4_ is yellow, while that of HCNM3 changes to a gray-green, which is consistent with the absorption spectra of the samples. The incorporation of Mo species also alters the band structure of the HCNM3 catalyst. The UV–vis DRS indicates that the corresponding bandgap energies (Egs) for the HCNM3 and g-C_3_N_4_ catalysts are 2.82 eV and 2.69 eV, respectively (Figure S15).

The photoluminescence (PL) spectra were utilized to investigate the separation and recombination efficiency of charge carriers (Figure 4b). The PL intensity of the CNM composites was significantly weaker than that of g-C_3_N_4_. The quenching of PL essentially indicates faster interfacial charge transport, which may be attributed to structural and morphological optimization, as well as the more metallic nature of Mo species facilitating electron relocalization to impede charge recombination. To further explore the charge–carrier separation/recombination in HCNM3, time-resolved PL decay spectra were also examined. As depicted in Figure 4c, the average lifetime of photoexcited carriers for HCNM3 was prolonged to 1.99 ns compared to 1.46 ns for g-C_3_N_4_ [58,59]. This result further confirms the higher rate of photogenerated charge separation.

**Figure 5:**
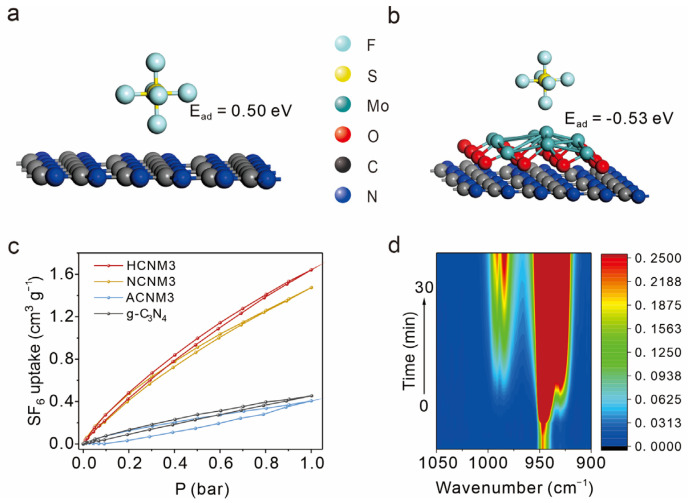
The adsorption and activation of SF_6_. (**a**) The g-C_3_N_4_-SF_6-ads_ structure with its adsorption energy by DFT calculation, (**b**) the MoO_2_-C_3_N_4_-SF_6-ads_ structure with its adsorption energy by DFT calculation, (**c**) SF_6_ sorption isotherms at 273 K of g-C_3_N_4_ and CNMs, (**d**) In situ FT-IR spectra recorded after the adsorption of SF_6_.

To further explore the mechanism of adsorption, DFT calculation is used to study the surface charge transfer and the absorption process between the MoO_2_, g-C_3_N_4_, CNM, and SF_6_ structures. After SF_6_ adsorption on the MoO_2_ or g-C_3_N_4_ interface, their adsorption energies are 0.17 eV and 0.50 eV, respectively, which means that the MoO_2_ and g-C_3_N_4_ interfaces do not have adsorption properties for SF_6_ (Figure 5a and Figure S16). However, as shown in Figure S17, electrons transfer from MoO_2_ to C_3_N_4_ after forming a heterostructure. It results in more positive charges on MoO_2_ to adsorb SF_6_. As a result, the adsorption energy of MoO_2_-C_3_N_4_ to SF_6_ is −0.53 eV (Figure 5b).

To further substantiate the adsorption performance of the materials towards SF_6_, pressure swing SF_6_ adsorption/desorption and spectroscopic studies were conducted. Given the large porosity and the abundance of Mo atoms, which serve as adsorption sites for SF_6_, HCNM3 and NCNM3 act as more effective SF_6_ adsorbents with capacities of 1.6 cm^3^/g and 1.5 cm^3^/g, respectively, compared to g-C_3_N_4_ (0.45 cm^3^/g) at 273 K and 1 atm. Despite its smaller surface area, the modified ACNM3 exhibits an uptake of SF_6_ of 0.41 cm^3^/g at 273 K and 1 atm, which is comparable to g-C_3_N_4_, and a similar trend was also detected on the sulfur hexafluoride adsorption curve at 298 K 1 atm (Figure 5c and Figure S18).

In situ FT-IR further observed the adsorption behavior of SF_6_ on the g-C_3_N_4_ and HCNM3 catalysts (Figure 5d and Figure S19). The absorption peaks corresponding to SF_6_ adsorption are located in the 900–1000 cm^−1^ range. For ease of comparison, peaks in the range of 970–1000 cm^−1^ were analyzed. On HCNM3, the signal corresponding to SF_6_ showed a gradual increase over time accompanied by a red-shift, indicating the adsorption and activation of SF_6_ gas molecules on the HCNM3 surface. In contrast, g-C_3_N_4_ quickly reached adsorption saturation after gas introduction, with peak positions being more positive and peak intensities weaker than those of HCNM3. This semi-quantitative result indicates that, compared to g-C_3_N_4_, HCNM3 has a stronger adsorption and activation effect on SF_6_ and can adsorb a greater amount of SF_6_, which is consistent with the SF_6_ adsorption experiments and DFT calculation results.

**Figure 6:**
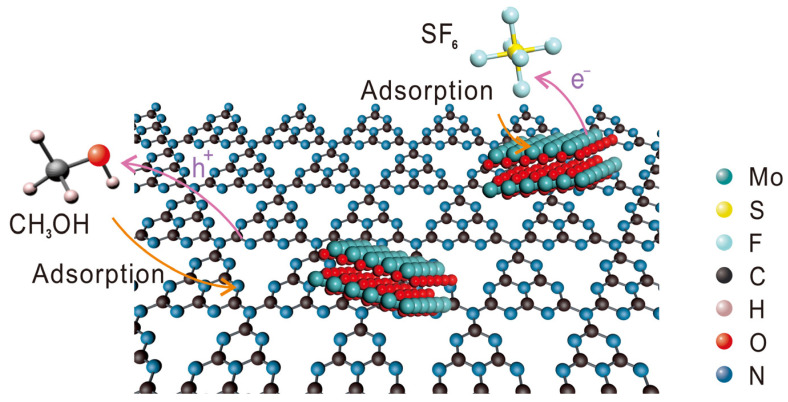
Schematic illustration of the photocatalytic reaction pathway for SF_6_.

We also conducted analyses on the substances present in the system after degradation. The signal peak at approximately −190 ppm in the nuclear magnetic resonance spectroscopy (NMR) spectrum revealed the generation of fluoride ions in the reaction solution (Figure S20), with no detection of C–F bond formation. F^−^, SO_3_^2−^, and SO_4_^2−^ were detected via ion chromatography (IC) as the main reaction products in the solution (Figure S21). Gas chromatography did not detect any new substances. Thus, it can be inferred that the main products of the SF_6_ photoreduction are F^−^, SO_3_^2−^, and SO_4_^2−^. Based on these findings, we propose a photocatalytic reaction pathway for SF_6_ (Figure 6): The MoO_x_ portion of the catalyst, which has a stronger adsorption affinity for SF_6_, initially adsorbs and activates the S–F bond in SF_6_. Upon light irradiation, the catalyst absorbs photons and separates into photoinduced electron–hole pairs. Subsequently, the electrons are transferred to SF_6_. Methanol acts as a sacrificial agent for the holes, as it is oxidized on the organophilic CN substrate, enabling the continuous separation of electron–hole pairs. Ultimately, the fluorine in SF_6_ is converted into F^−^/HF, while the sulfur element is transformed into SO_x_^2−^ species.

## 3. Materials and Methods

### 3.1. Materials

Methanol and acetonitrile were purchased from Aladdin Industrial Corporation (Beijing, China). Ammonium molybdate tetrahydrate ((NH_4_)_6_Mo_7_O_24_·4H_2_O) and melamine were sourced from Grat Company (Wuhan, China). SF_6_ was acquired from Shanghai Weichuang Standard Gas Company Limited (Shanghai, China). All reagents were used as received without further purification. Milli-Q water (Millipore Corp., Burlington, MA, USA, with a resistivity of 18.2 MΩ·cm) was employed for the experiments.

### 3.2. Preparation of g-C_3_N_4_

g-C_3_N_4_ was synthesized by heating melamine at 550 °C for 4 h with a heating rate of 2 °C/min in a muffle furnace.

### 3.3. Preparation of CNMs

The CNM samples were prepared by directly mixing and griding g-C_3_N_4_ powders (2 g) with different amounts (0.26, 0.77, and 1.29 g, respectively) of (NH_4_)_6_Mo_7_O_24_·4H_2_O powders. Then, they were placed in a crucible with a cover for re-polymerization. Next, they were placed in a quartz tube furnace with 5% H_2_/Ar and N_2_, followed by a muffle furnace at 550 °C for another 2 h, respectively. After being naturally cooled down, the collected samples were denoted as HCNMx, NCNMx, and ACNMx, correspondingly, where x represents the initial amount of (NH_4_)_6_Mo_7_O_24_·4H_2_O (x = 1, 3, and 5). The detailed information is shown in Table 2.

### 3.4. Photocatalytic SF_6_ Degradation Experiments

The photocatalytic reduction reactions of SF_6_ were conducted in a Schlenk flask reactor (40 mL). In the Schlenk flask, 30 mg of the catalyst was dispersed in a mixture containing 0.5 mL of methanol and 2.5 mL of solvent (acetonitrile: 2 mL; H_2_O: 0.5 mL). Prior to irradiation with a 50 W 365 nm UV-LED lamp (Epileds, Taiwan, China), the reaction vessel was repeatedly evacuated to completely remove air and then filled with SF_6_/Ar gas (1 atm, ~5% SF_6_). During the photocatalytic reaction, the reaction mixture was vigorously stirred at room temperature. For the recycling experiments, the reactor was refilled with the same gas mixture. After the reaction, the performance was assessed using gas chromatography.

### 3.5. Density Functional Theory (DFT) Computations

The DFT calculation was performed by using plane-wave basis sets on Materials Studio 2020 version. In order to optimize the MoO_2_, g-C_3_N_4_, CNM, and SF_6_ structures, the exchange correlation function, PBE, generalized gradient approximation with Koelling–Hamon relativistic treatment, and spin polarization assumptions were employed. Broyden–Fletcher–Goldfarb–Shanno geometry optimization is used for cell optimization. The interaction between valence electrons and the ionic core is described by using On-The-Fly-Generation ultra soft pseudo potential. The kinetic cutoff energy for the convergence test is 400 eV, and a k-point set mesh (1 × 1 × 1) parameter is used for Brillouin zone sampling. The threshold for self-consistent field iterations used is 5.0 × 10^−6^ eV atom^−1^. The convergence tolerance parameters of the optimized calculation are the tolerance for energy 2.0 × 10^−5^ eV atom^−1^, the maximum force of 0.05 eV Å^−1^, and the maximum displacement of 2 × 10^−3^ Å.

The adsorption performance of MoO_2_, g-C_3_N_4_, and CNM is investigated using E_ads_, where E_ads_ is the adsorption energy of the SF_6_ molecule on the different structures. The E_ads_ is calculated using the following equation:

E_ads_ = E_SF6 molecule_ − E_material_ − E_SF6 molecule_

where E_SF6 molecule_, E_material_, and E_SF6 molecule_ are the energies of the SF_6_ material, material, and SF_6_ molecule, respectively.

### 3.6. Characterization

The quantity of SF_6_ was ascertained utilizing gas chromatography (GC 7900, Techcomp Instrument Co., Ltd., Shanghai, China) equipped with a thermal conductivity detector (TCD). The operational temperatures for the column, injection port, and detector were maintained at 100, 100, and 150 °C, correspondingly. The specimen’s crystallographic information was captured via X-ray diffraction (XRD) on a Bruker D8 Advance diffractometer (Billerica, MA, USA) employing Cu-Kα radiation (λ = 1.5406 Å). Vibrational characteristics were probed with Raman spectroscopy on an inVia Qontor Renishaw instrument (Gloucestershire, UK). The physical configuration of the specimen was scrutinized using scanning electron microscopy (SEM, Nova NanoSEM 450, FEI, Hillsboro, OR, USA) operated at an accelerating voltage of 10 kV. The elemental distribution within the specimen was delineated via transmission electron microscopy (TEM, FEI Talos F200X) functioning at 200 kV in conjunction with Fischione model 2550 for specimen preparation. The specimen’s valence and electronic configurations were determined via X-ray photoelectron spectroscopy (XPS, AXIS Ultra DLD, Shimadzu, Kyoto, Japan), with Al Kα radiation serving as the excitation source. The optical absorption properties of the specimens were evaluated through UV/Vis diffuse reflectance spectroscopy on a Lamda 950 spectrophotometer (Perkin-Elmer, Waltham, MA, USA). Liquid samples were filtered for ion chromatography (IC) measurement on a Thermo Fisher ICS5000+ system (Waltham, MA, USA). For multivalent anions, an AS11/AG11 column set with NaOH eluent (1 mL/min) and conductivity detection was used; sulfate (S_x_O_y_^2−^) employed AS11-HC/AG11-HC columns with the EGC-generated KOH gradient (1 mL/min).

## 4. Conclusions

In summary, we have successfully developed a thin-layer CN material loaded with MoO_x_ (CNM) for the efficient degradation of SF_6_, a potent greenhouse gas with significant environmental implications. The photocatalytic performance of the CNM was outstanding, achieving a degradation efficiency for SF_6_ of up to 1.73 mmol/g after one day of LED light irradiation at room temperature, which is notably more than eight times higher than that of pristine g-C_3_N_4_ (0.21 mmol/g). This remarkable enhancement can be attributed to the synergistic effects of the high specific surface area of the thin-layer CN, the introduction of MoO_2_ active sites, and the efficient electron transport facilitated by the Mo-N bonds. The combination of high specific surface area materials with Mo-based inorganic nanocatalytic materials presents a promising strategy for enhancing photocatalytic activity, which could be further explored for the degradation of other environmentally harmful substances. The findings of this study pave the way for low-energy, high-efficiency solutions to mitigate the impact of potent greenhouse gases like SF_6_, contributing to global efforts in combating climate change.

## Figures and Tables

**Table 1:** nC/nN of g-C_3_N_4_, HCNM3, NCNM3, and ACNM3 were obtained through simulation calculations, as depicted in Figure 2.

Samples	nC/nN
HCNM3	0.97
NCNM3	1.1
ACNM3	3.6
g-C_3_N_4_	0.97

**Table 2:** Detailed information of CNM preparation.

	Atmosphere	5% H_2_/Ar	N_2_	Air
MMo (g)				
0.26 g		HCNM1	NCNM1	ACNM1
0.77 g		HCNM3	NCNM3	ACNM3
1.29 g		HCNM5	NCNM5	ACNM5

## Data Availability

Data will be made available upon request.

## Supplementary Materials

The following supporting information can be downloaded at https://www.mdpi.com/article/10.3390/molecules30071481/s1. References [60,61,62,63] are cited in the supplementary materials.

## Abbreviations

The following abbreviations are used in this manuscript:

CNM	carbon nitride material loaded with MoO_x_
GWP	global warming potential
SF_6_	sulfur hexafluoride
in situ IR	in situ infrared spectroscopy
DFT	Density Functional Theory
XPS	X-ray photoelectron spectroscopy
XRD	X-ray diffraction
TEM	Transmission electron microscope
FTIR	Fourier transform infrared
DRS	diffuse reflectance spectra
SEM	scanning electron microscope
IC	ion chromatography
NMR	nuclear magnetic resonance spectroscopy
GC	gas chromatography
TCD	thermal conductivity detector
SAED	selected area electron diffraction
