# Supplementary material for: Surface-Engineered MoOx/CN Heterostructures Enable Long-Term SF6 Photodegradation via Suppressed Fluoridation
Source: Molecules. 2025 Mar 27;30(7):1481. doi: 10.3390/molecules30071481 (PMC11990455; doi:10.3390/molecules30071481)
Supplement: Supplementary file 1 [file molecules-30-01481-s001.zip › molecules-3515763-supplementary.pdf]

Supporting Information

Surface-Engineered MoOx/CN Heterostructures Enable Long-Term SF6 Photodegradation via Suppressed Fluoridation

Wenhui Zhou †, Boxu Dong † , Ziqi Si, Yushuai Xu, Xinhua He, Ziyi Zhan, Yaru Zhang, Chaoyu Song, Zhuoqian Lv, Jiantao Zai * and Xuefeng Qian *

Shaoxing Research Institute of Renewable Energy and Molecular Engineering, School of Chemistry and Chemical Engineering, Shanghai Jiao Tong University, Shanghai 200240, China; zhouwenhui1995@sjtu.edu.cn (W.Z.); kingjames@sjtu.edu.cn (B.D.); siziqi520@163.com (Z.S.); yushuai-xu@sjtu.edu.cn (Y.X.); xinhuahe@sjtu.edu.cn (X.H.); joieyzhan@gmail.com (Z.Z.); fishzyrrr@sjtu.edu.cn (Y.Z.); songchaoyu@sjtu.edu.cn (C.S.); lzq323@sjtu.edu.cn (Z.L.)

* Correspondence: zaijiantao@sjtu.edu.cn (J.Z.); xfqian@sjtu.edu.cn (X.Q.)

† These authors contributed equally to this work.

Supplementary Method

Experimental method for fluorescence lifetime determination

Data Collection: Use a time-correlated single photon counting (TCSPC) setup or a similar technique to collect fluorescence decay data. This will give you a histogram of photon counts versus time, representing the fluorescence decay profile.

Fitting data: We can perform the fitting of fluorescence lifetime using the software provided with the fluorescence instrument or the Origin software by following equation.

$$I(t) = \sum_{i=1}^{n} A_i \exp\left(-\frac{t}{\tau_i}\right)$$

where A_i are the amplitudes and τ_i are the lifetimes of the individual components.

The fitting range spans from the maximum point of the intensity (I) to the point where the intensity (I) decreases to equilibrium.

In this study, the data were fitted using a third-order model.

Based on the fitting results obtained for A_i and τ_i, the average lifetime can be calculated as follows:

$$\langle\tau\rangle_{\text{intensity-weighted}} = \frac{\sum_{i=1}^{n} A_i \tau_i^2}{\sum_{i=1}^{n} A_i \tau_i}$$

Goodness of Fit: Assess the quality of the fit using statistical measures such as the chi-squared (χ^2) value, residuals, and visual inspection of the fit. A good fit should have a χ^2 value close to 1 and randomly distributed residuals.

Supplementary Figures

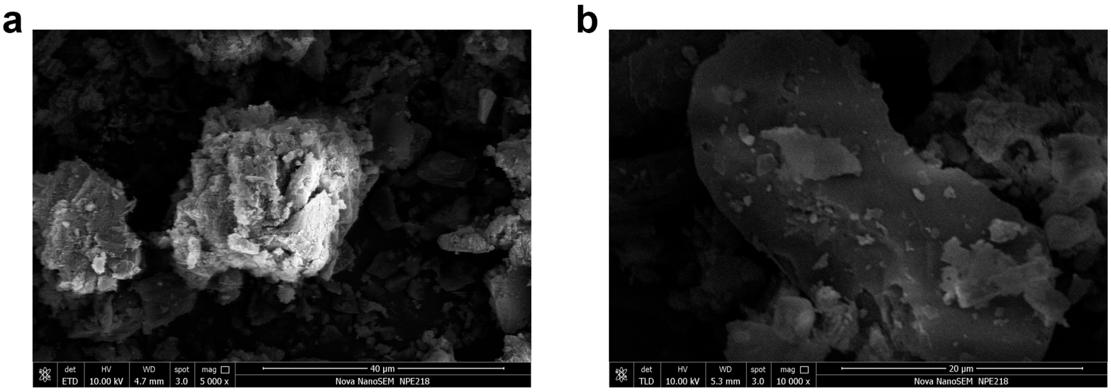

Figure S1. Scanning electron microscope (SEM) images of HCNM3.

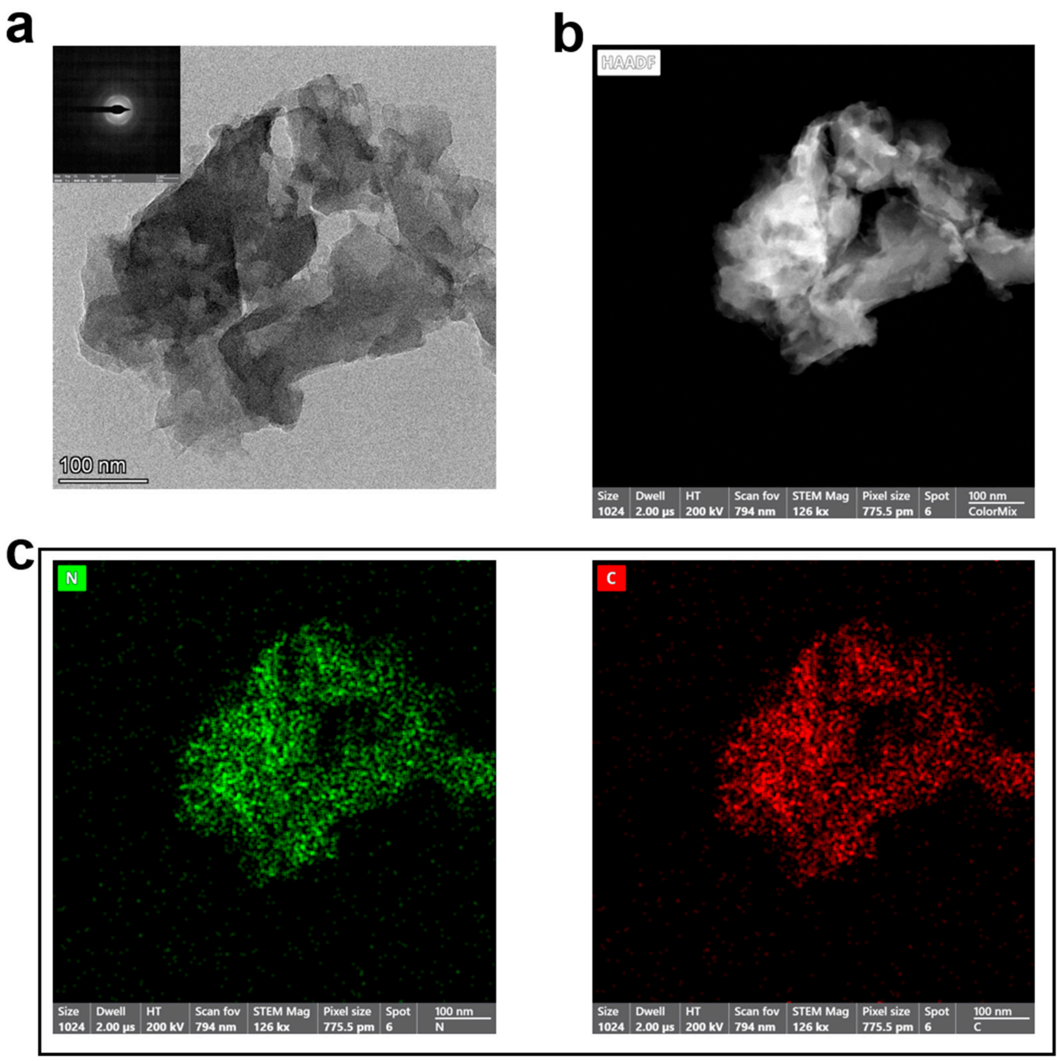

Figure S2. (a)transmission electron microscope (TEM), insert (a) was Selected area electron diffraction (SAED), (b) HAADF-STEM (scanning transmission electron microscope) images and (c) STEM-EDS maps of g-C_3N_4.

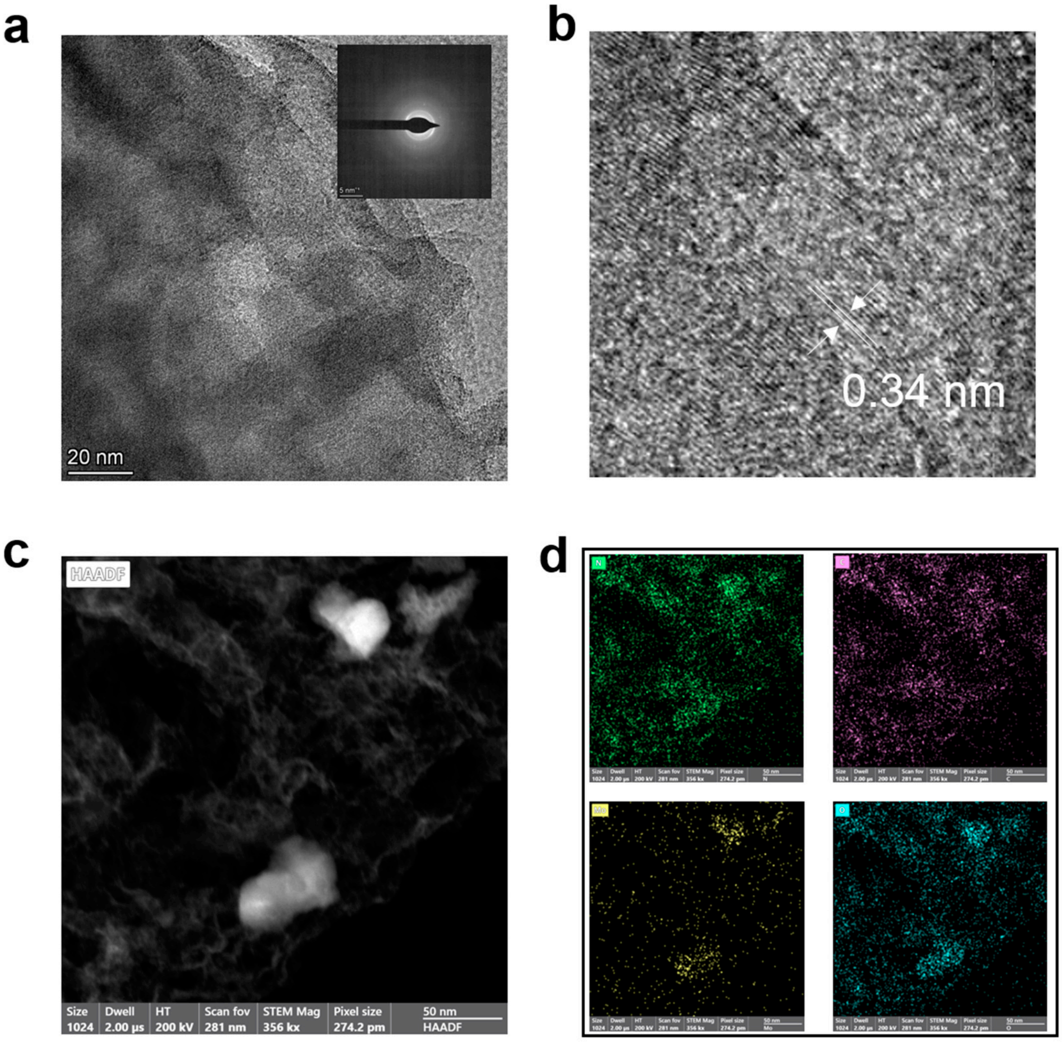

Figure S3. (a) HRTEM, (b) HRTEM images in the box area of (a), (c)HAADF-STEM (scanning transmission electron microscope) images, and (d) STEM-EDS maps of HCNM3.

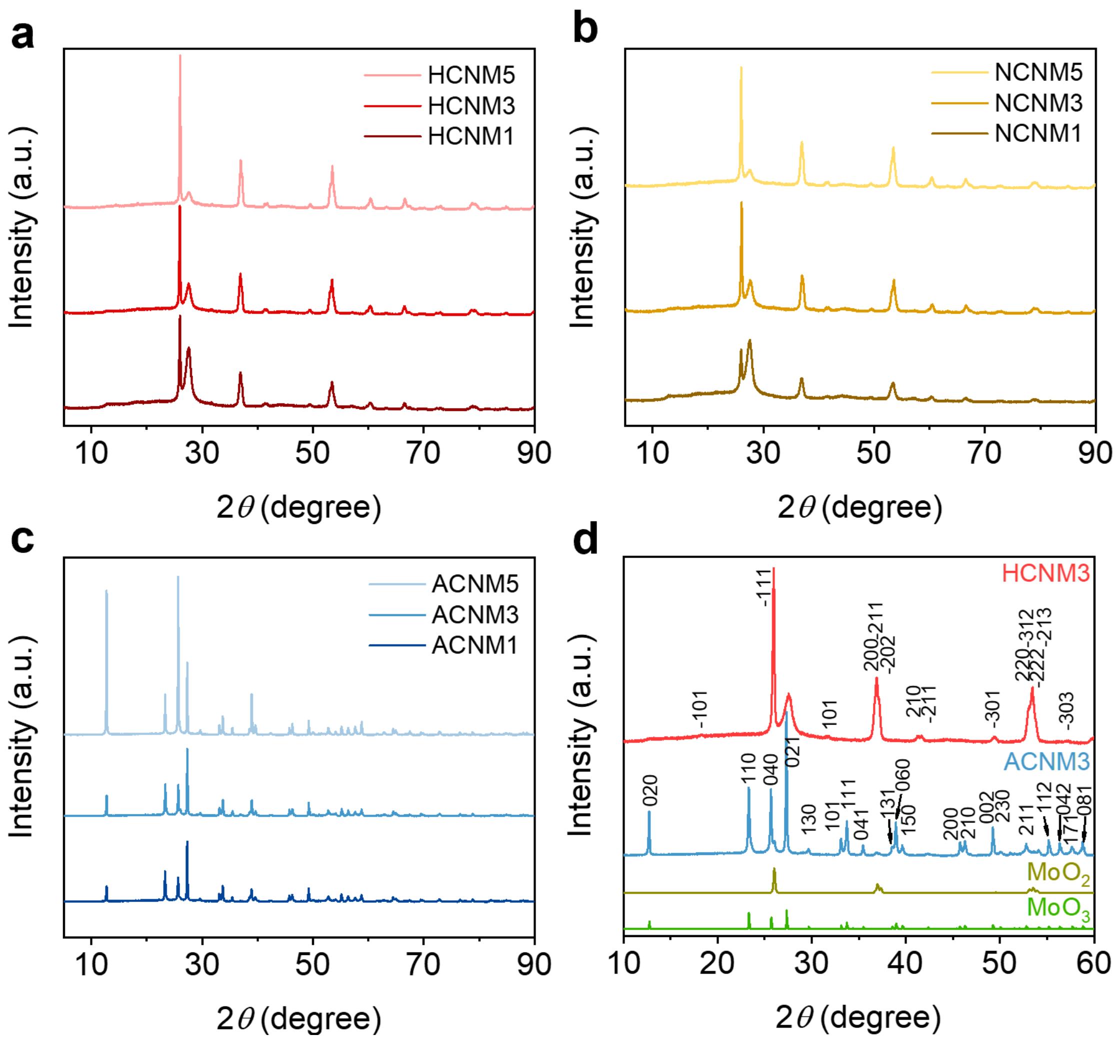

Figure S4. X-ray diffraction (XRD) patterns of (a) HCNMs, (b) NCNMs, (c)ACNMs, and (d) HCNM3, ACNM3, MoO_2 (JCPDS: 32-0671), and MoO_3 (JCPDS: 50–0508).

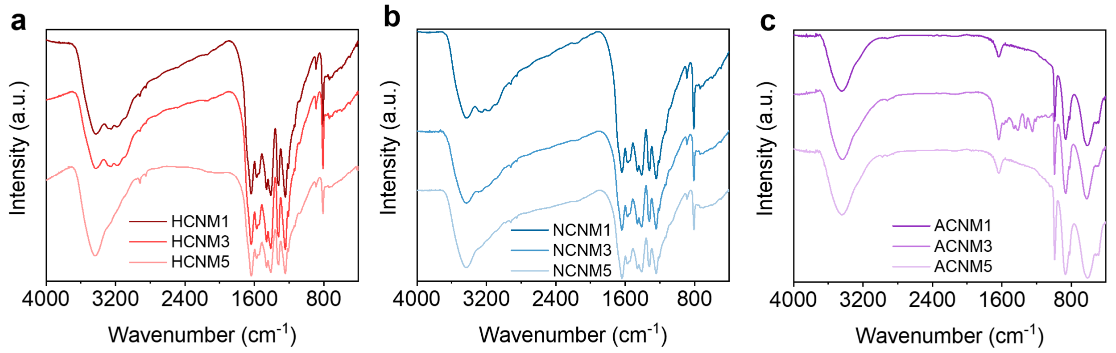

Figure S5. FTIR spectra of (a) HCNMs, (b) NCNMs, and (c)ACNMs.

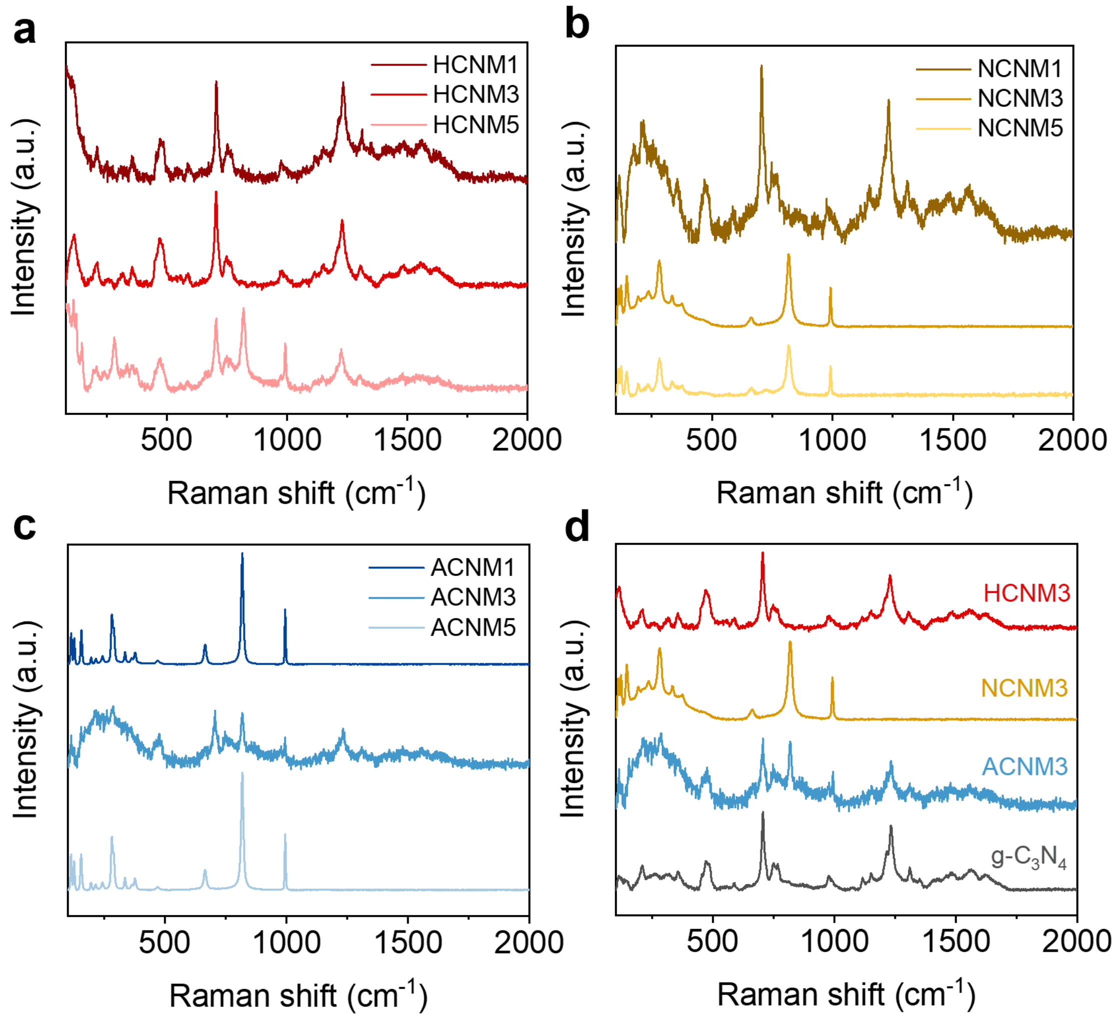

Figure S6. Raman spectroscopy of (a) HCNMs, (b) NCNMs, (c)ACNMs, and (d) g-C_3N_4 and CNMs.

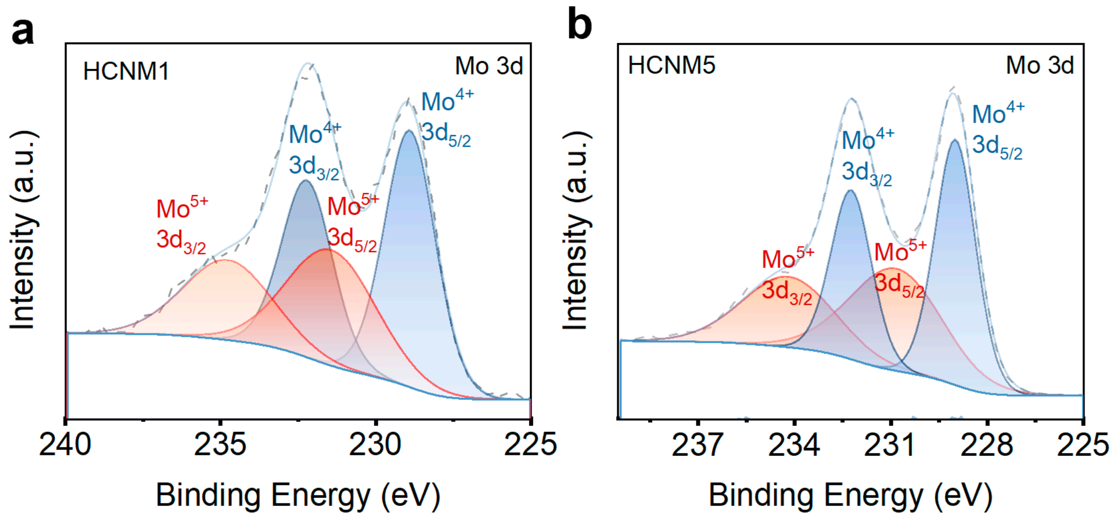

Figure S7. High-resolution Mo 3d XPS spectra of (a) HCNM1, and (b) HCNM5.

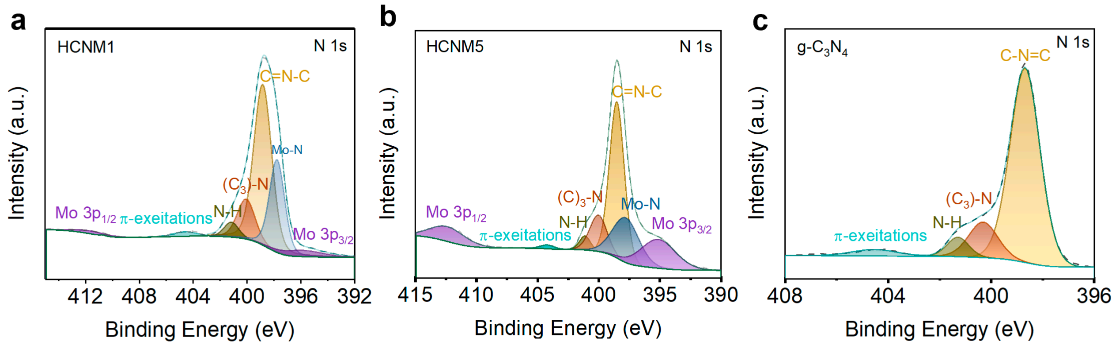

Figure S8. High-resolution N 1s XPS spectra of (a) HCNM1, (b) HCNM5, and (c) g-C_3N_4.

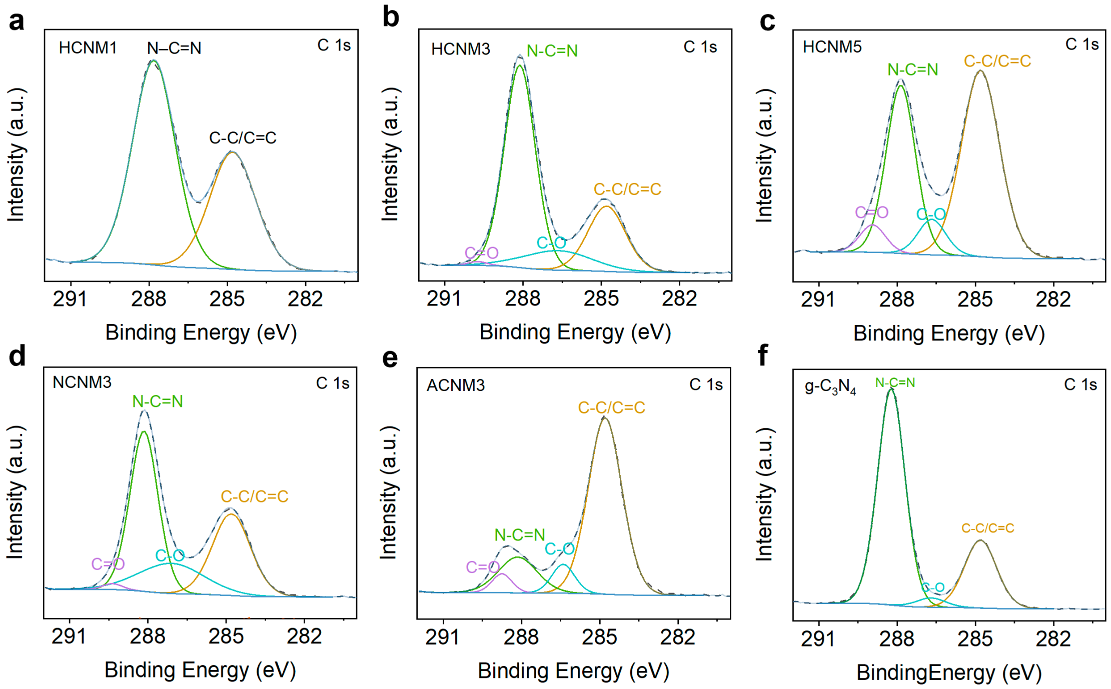

Figure S9. High-resolution C 1s XPS spectra of (a) HCNM1, (b) HCNM3, (c) HCNM5, (d) NCNM3, (e) ACNM3, and (f) g-C_3N_4.

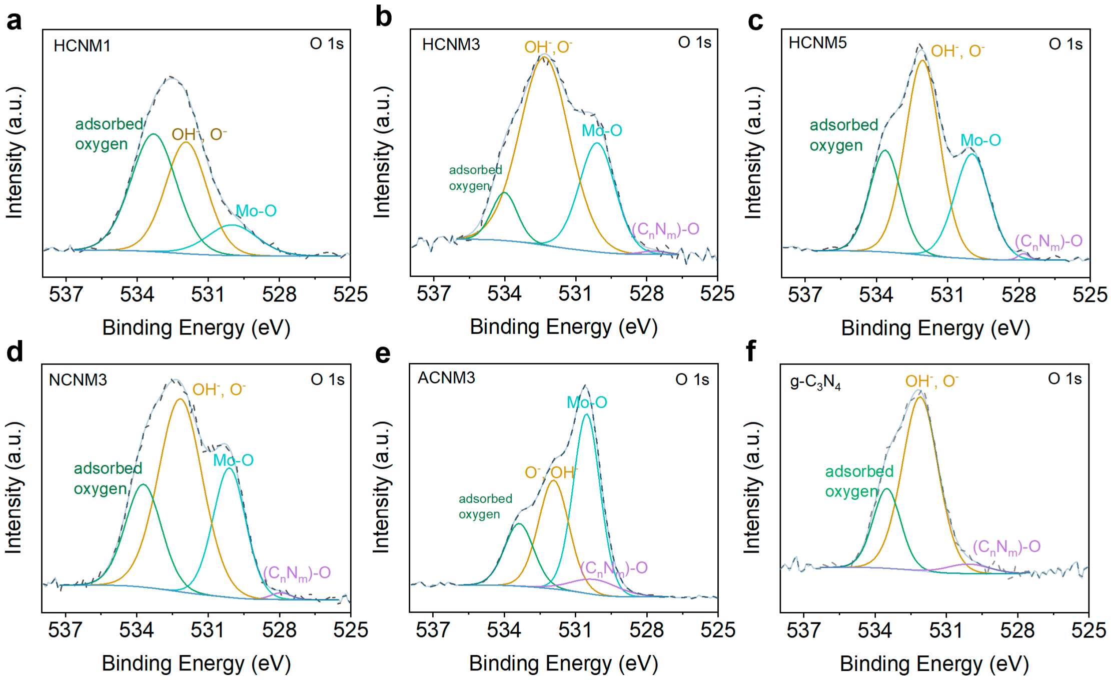

Figure S10. High-resolution O 1s XPS spectra of (a) HCNM1, (b) HCNM3, (c) HCNM5, (d) NCNM3, (e) ACNM3, and (f) g-C_3N_4.

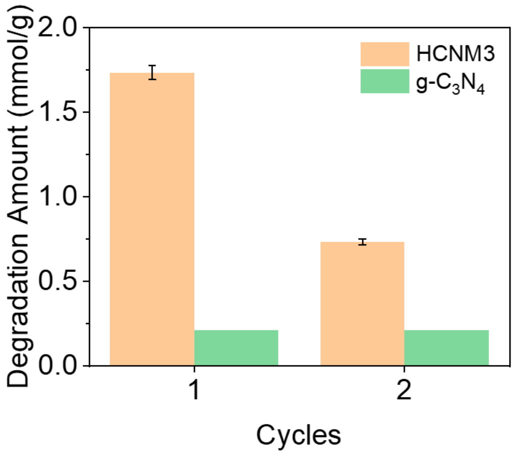

Figure S11. SF_6 degradation amount in the stability tests of HCNM3 and g-C_3N_4 sample with the reaction hours of 24h.

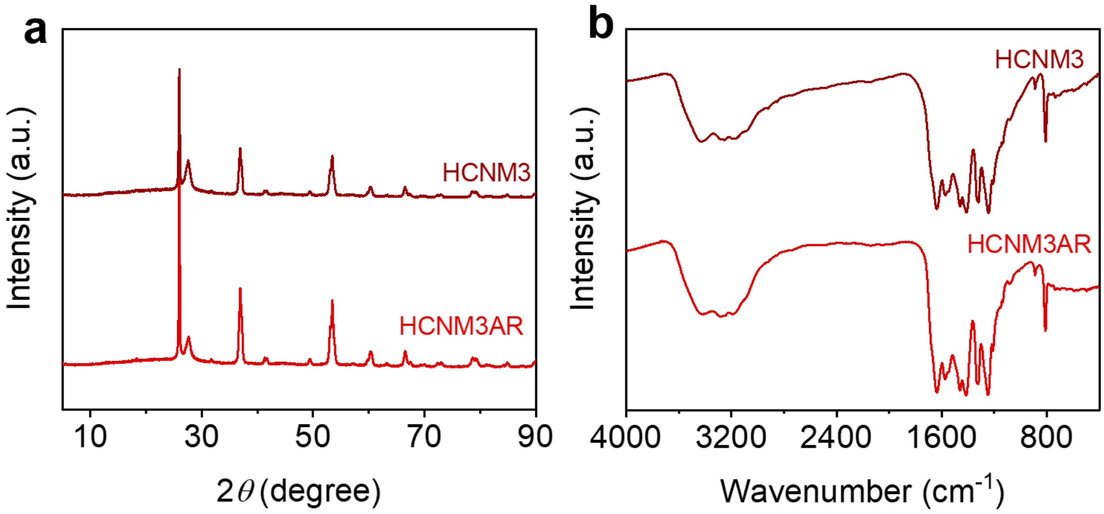

Figure S12. (a)XRD patterns and (b) FTIR spectra of HCNM3 after reaction.

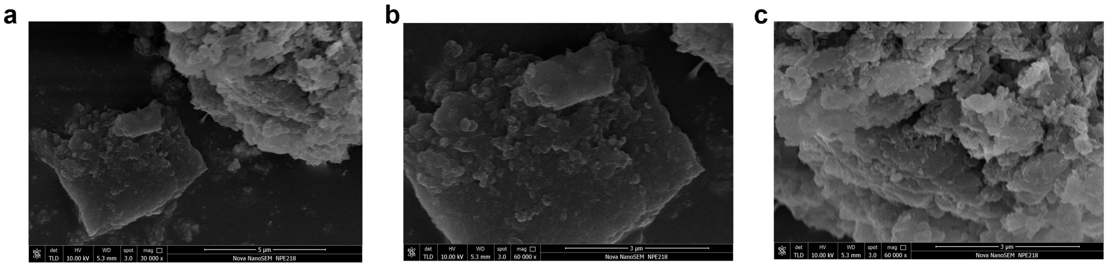

Figure S13. SEM images of HCNM3 after reaction.

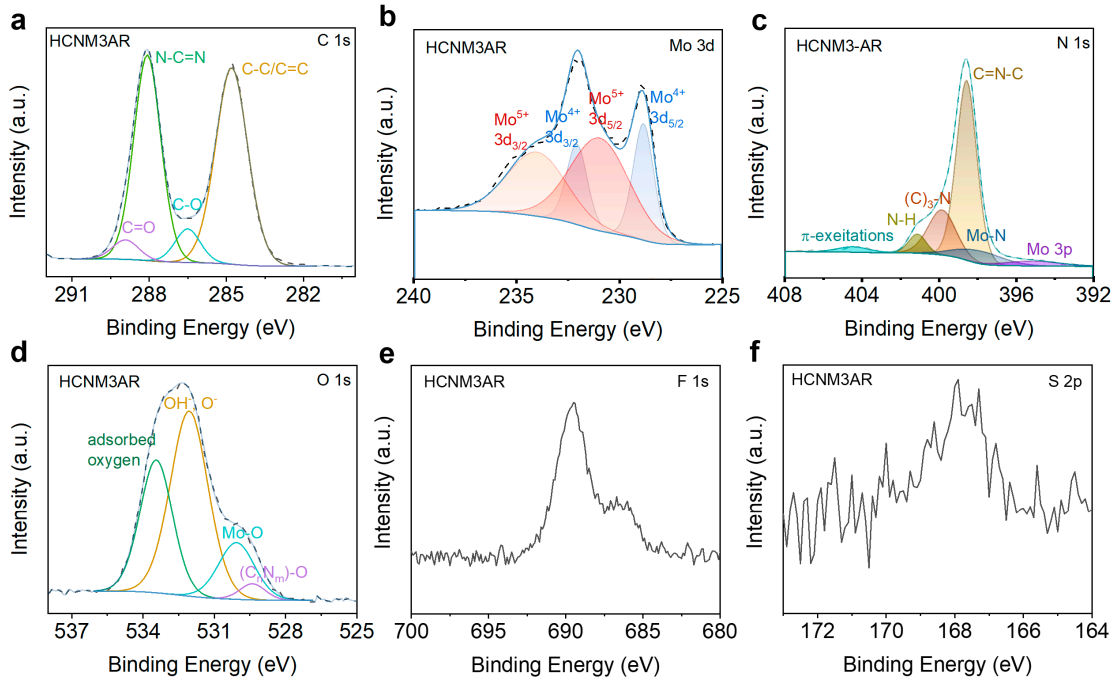

Figure S14. High-resolution (a) C 1s, (b) Mo 3d, (c) N 1s, (d) O 1s, (e) F 1s, and (f) S 2p XPS spectra of HCNM3 after reaction.

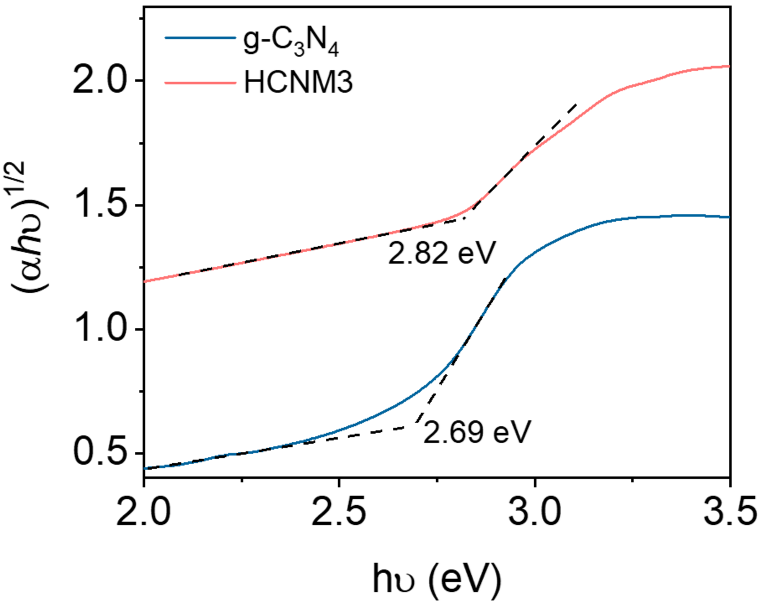

Figure S15. Optical band gaps of g-C_3N_4 and HCNM3 determined by UV-vis diffuse reflectance spectra (DRS).

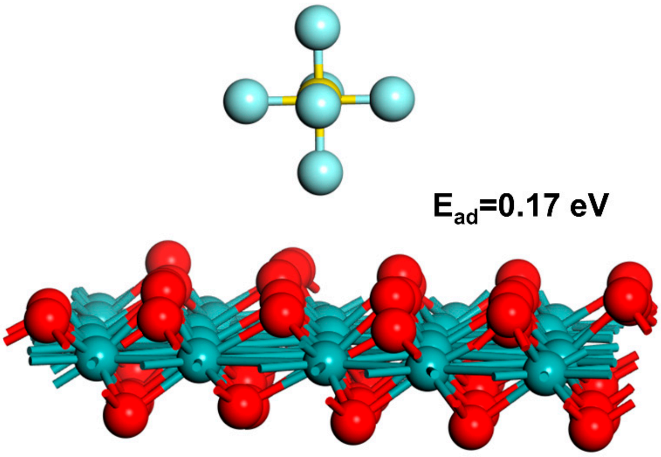

Figure S16. MoO_2-$SF_{6\text{-}ads}$ structure with its adsorption energy by DFT (Density Function Theory) calculation.

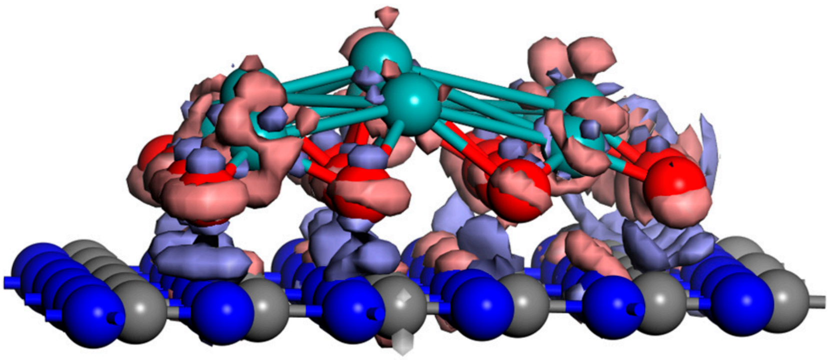

Figure S17. Charge-density differences at the interface of the MoO_2-C_3N_4 structure (iso-surface: 0.05 a.u.; purple color: electron accumulation; red color: electron depletion).

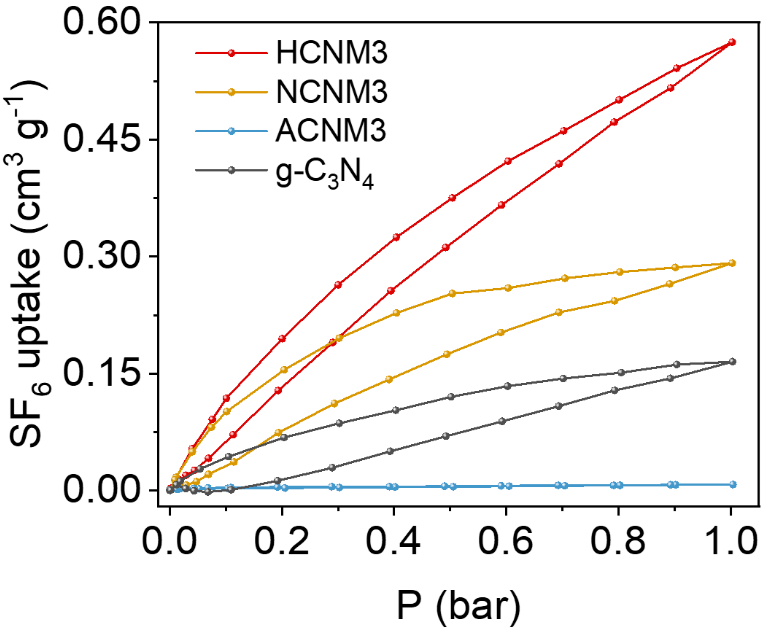

Figure S18. SF_6 sorption isotherms at 298 K of g-C_3N_4 and CNMs.

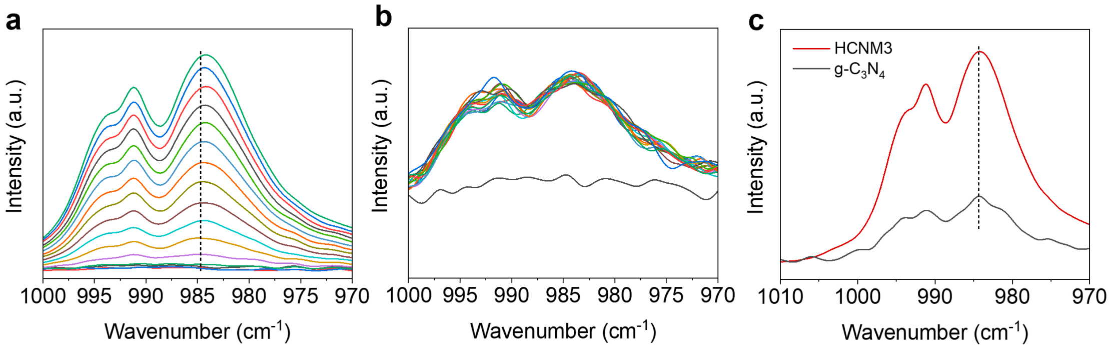

Figure S19. *In-situ* infrared detection of SF_6 molecule behavior on (a) HCNM3, and (b) g-C_3N_4, and (c) comparison of them at 30 minutes.

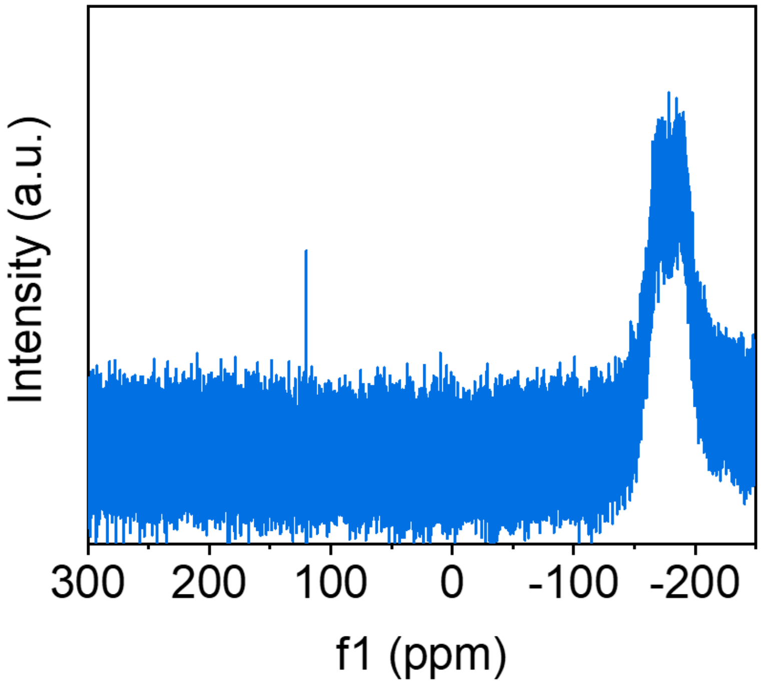

Figure S20. ^{19}F NMR spectrum of reaction solutions.

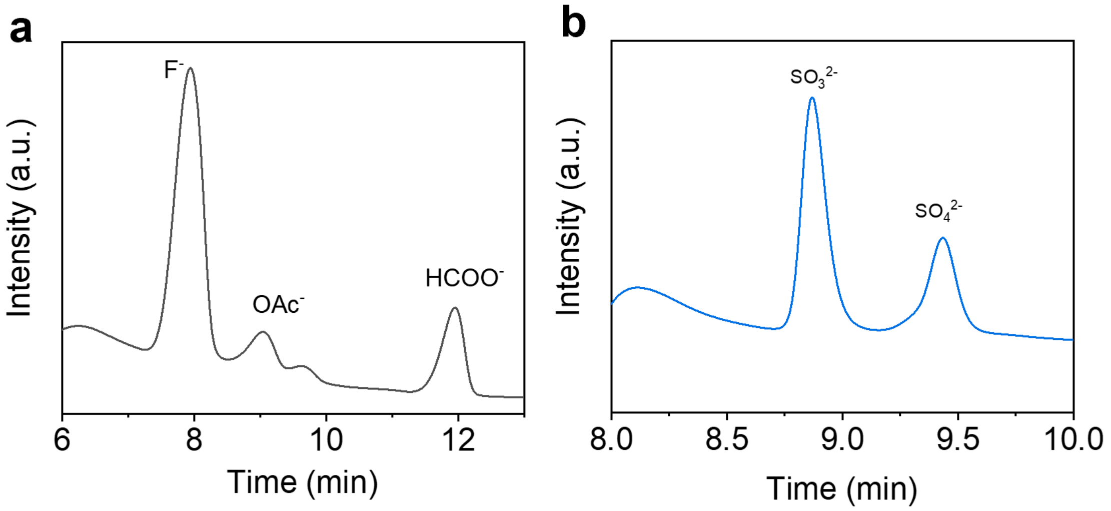

Figure S21. Ion chromatogram for cations of reaction solutions.

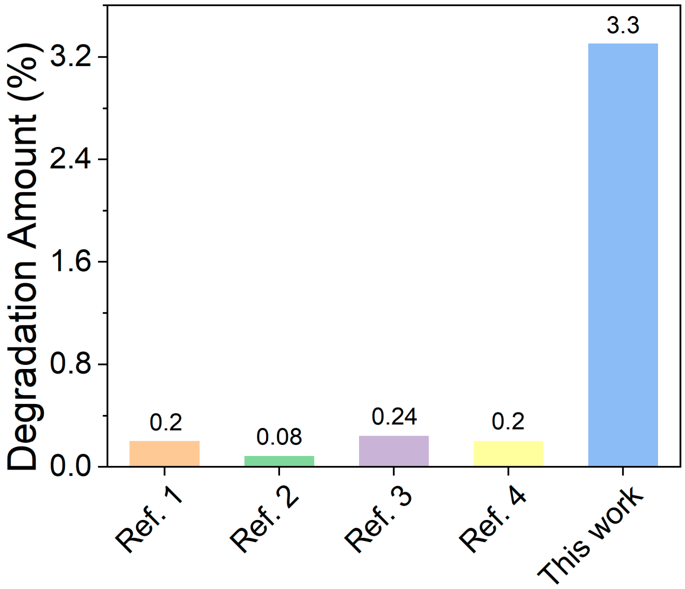

Figure S22. Comparison of photocatalytic SF_6 degradation efficiency of previously reported work and this work. Ref.1: J. Environ. Sci., 20 (2008) 183-188.[1] Ref.2: J. Hazard. Mater., 168 (2009) 493-500.[2] Ref.3: Chemosphere, 66 (2007) 833-840.[3] Ref.4: J. Hazard. Mater., 151 (2008) 323-330.[4]

References:

[1] Huang, L.; Gu, D. H.; Yang, L. Y.; Xia, L. Y.; Zhang, R. X.; Hou, H. Q. Photoreductive degradation of sulfur hexafluoride in the presence of styrene. *J Environ Sci* **2008**, *20* (2), 183-188. DOI: Doi 10.1016/S1001-0742(08)60029-7.

[2] Song, X. X.; Liu, X. G.; Ye, Z. L.; He, J. C.; Zhang, R. X.; Hou, H. Q. Photodegradation of SF_6 on polyisoprene surface: Implication on elimination of toxic byproducts. *J Hazard Mater* **2009**, *168* (1), 493-500. DOI: 10.1016/j.jhazmat.2009.02.047.

[3] Huang, L.; Dong, W. B.; Zhang, R. X.; Hou, H. Q. Investigation of a new approach to decompose two potent greenhouse gases: Photoreduction of SF_6 and SF_5CF_3 in the presence of acetone. *Chemosphere* **2007**, *66* (5), 833-840. DOI: 10.1016/j.chemosphere.2006.06.028.

[4] Huang, L.; Shen, Y.; Dong, W. B.; Zhang, R. X.; Zhang, J. L.; Hou, H. Q. A novel method to decompose two potent greenhouse gases:: Photoreduction of SF_6 and SF_5CF_3 in the presence of propene. *J Hazard Mater* **2008**, *151* (2-3), 323-330. DOI: 10.1016/j.jhazmat.2007.05.080.
